# Assessing the Relationship Between Sleep Duration and Quality and Mental Health Among University Students

**DOI:** 10.1192/bjo.2025.10079

**Published:** 2025-06-20

**Authors:** Hamid Alhaj, Amin Al Borom, Yosser Al-Sadoon, Deena Al Amad, Alya Alzaabi

**Affiliations:** College of Medicine, University of Sharjah, Sharjah, UAE

## Abstract

**Aims:** Despite sleep being an important factor in maintaining mental health, some students frequently experience poor sleep quality and insufficient duration. Reasons such as prioritising academic duties over sleep and well-being may be implicated in the understanding of this relationship. Our study aims to assess the relationship between sleep duration and quality and mental health parameters among university students in the UAE.

**Methods:** A cross-sectional study was conducted with 550 university students, of whom 420 met inclusion criteria. Most notably, 81 were excluded due to pre-existing medical conditions. Data was collected using an online self-administered questionnaire incorporating the Generalized Anxiety Disorder-7 (GAD-7), Patient Health Questionnaire-9 (PHQ-9), and Pittsburgh Sleep Quality Index (PSQI) to evaluate anxiety, depression, and sleep quality. Data was analysed using SPSS with significance at p < 0.05. Ethical approval from the University of Sharjah Ethics Committee was obtained, and informed consent was taken from all participants.

**Results:** The sample comprised 61% males (n=256) and 39% females (n=164), predominantly undergraduates (93.1%, n=391) aged 18–23 (92.9%, n=390). Poor sleep quality (PSQI≥5) was identified in 87.2% (n=366), with a mean PSQI score of 7.84 (SD=3.26). Mean anxiety (GAD-7) and depression (PHQ-9) scores were 8.49 ± 5.655 and 9.75 ± 6.376, respectively, with pronounced gender disparities: 24.6% of females reported severe anxiety symptoms versus 6.7% of males (p<0.001). Similarly, females scored higher on PHQ-9, with 25.8% having severe depressive symptoms compared with 12.8% of males (p=0.004). Mid-range GPA students (3.0–3.5) had significantly worse sleep quality (PSQI = 8.75±3.73) compared with higher (3.5–4.0; 7.22±2.88) and lower (<3.0; 7.84±3.10) GPA groups (p = 0.003). Participants with severe anxiety had significantly higher PSQI scores (10.28±3.362) than those with none/mild symptoms (6.98±2.863; p<0.001). Similarly, 81.1% with severe anxiety and 80.5% of students with severe depressive symptoms scored above the median PSQI score (median=7.00, p<0.001).

**Conclusion:** The study findings elucidate an important correlation between sleep quality, mental health parameters, and academic performance among university students, with notable gender disparities. Mid-range GPA students (3.0–3.5) reported the worst sleep quality, suggesting this group being more likely to experience stress-related sleep difficulties, while possibly striving for higher GPAs. The high prevalence of poor sleep, moderate-to-severe anxiety, and depression symptoms particularly among female students highlights a pressing need for gender-sensitive interventions. Further studies are required to ascertain culturally appropriate approaches to improve quality of sleep and mental health among university students.

*et al: Fatima Alameeri and Yousif Hameed

